# Radiomics prediction of MGMT promoter methylation in adult diffuse gliomas: a combination of structural MRI, DCE, and DTI

**DOI:** 10.3389/fneur.2025.1493666

**Published:** 2025-01-29

**Authors:** Yuying Liu, Zhengyang Zhu, Jianan Zhou, Han Wang, Huiquan Yang, Jinfeng Yin, Yitong Wang, Xin Li, Futao Chen, Qian Li, Zhuoru Jiang, Xi Wu, Danni Ge, Yi Zhang, Xin Zhang, Bing Zhang

**Affiliations:** ^1^Department of Radiology, Nanjing Drum Tower Hospital, Affiliated Hospital of Medical School, Nanjing University, Nanjing, China; ^2^National Key Laboratory for Novel Software Technology, Department of Computer Science and Technology, Nanjing University, Nanjing, China; ^3^Nanjing Center for Applied Mathematics, Nanjing, China; ^4^Department of Medical Imaging, Shandong Second Medical University, Jinan, China; ^5^Department of Medical Imaging, Shandong First Medical University, Jinan, China; ^6^Medical Imaging Center, Affiliated Drum Tower Hospital, Medical School of Nanjing University, Nanjing, China; ^7^Institute of Medical Imaging and Artificial Intelligence, Nanjing University, Nanjing, China; ^8^Jiangsu Key Laboratory of Molecular Medicine, Nanjing, China; ^9^Institute of Brain Science, Nanjing University, Nanjing, China

**Keywords:** MGMT, glioma, radiomics, DCE, DTI

## Abstract

**Purpose:**

To assess the predictive value of radiomics features extracted from structural MRI, dynamic contrast enhanced (DCE), and diffusion tensor imaging (DTI) in detecting O6-methylguanine-DNA methyltransferase (MGMT) promoter methylation in patients with diffuse gliomas.

**Methods:**

Retrospective MRI data of 110 patients were enrolled in this study. The training dataset included 88 patients (mean age 52.84 ± 14.71, 47 females). The test dataset included 22 patients (mean age 50.64 ± 12.58, 12 females). A total of 2,782 radiomic features were extracted from structural MRI, DCE, and DTI within two region of interests (ROIs). Feature section was conducted using Pearson correlation and least absolute shrinkage and selection operator. Principal component analysis was utilized for dimensionality reduction. Support vector machine was employed for model construction. Two radiologists with 1 year and 5 years of experience evaluated the MGMT status in the test dataset as a comparison with the models. The chi-square test and independent samples *t*-test were used for assessing the statistical differences in patients’ clinical characteristics.

**Results:**

On the training dataset, the model structural MRI + DCE achieved the highest AUC of 0.906. On the test dataset, the model structural MRI + DCE + DTI achieved the highest AUC of 0.868, outperforming two radiologists.

**Conclusion:**

The radiomics models have obtained promising performance in predicting MGMT promoter methylation status. Adding DCE and DTI features can provide extra information to structural MRI in detecting MGMT promoter methylation.

## 1 Introduction

Gliomas are the most common primary malignant tumors of the central nervous system (CNS), characterized by significant heterogeneity in their biological behavior and clinical prognosis (1, 2). Currently, the standard treatment for gliomas includes neurological surgery followed by radiation and adjuvant chemotherapy with temozolomide (TMZ) (3, 4). The latest 2021 classification of CNS tumors highlighted the value of molecular markers in the clinical diagnosis and prognosis of gliomas (5, 6).

O6-methylguanine-DNA methyltransferase (MGMT) gene promoter methylation status is one of the critical molecular markers influencing the prognosis and treatment response in adult diffuse gliomas (7). MGMT is a DNA-repairing enzyme that counteracts the alkylating effects of TMZ by preventing DNA mismatches and apoptosis, reducing the cytotoxic effect of alkylating agents and resulting in tumor cells resistant to chemotherapy (8). When the MGMT promoter is methylated, the tumor cells are more susceptible to the cytotoxic effects of alkylating agents. Thus, patients with MGMT promoter methylation exhibit better therapeutic response with more prolonged progression-free survival (PFS) and overall survival (OS) compared to those without MGMT promoter methylation (9). Therefore, the accurate detection of MGMT methylation status is crucial for optimizing treatment plans and improvement of patient prognosis (10).

Traditionally, MGMT methylation status is assessed by sequencing the tumor tissue samples obtained from surgery or invasive biopsy, which is time-consuming and expensive. Clinically, MRI is commonly used for pre-operative diagnosis of gliomas. Non-invasive MRI-based radio-genomics provide a promising alternative for evaluating MGMT promoter methylation status before surgery. Structural MRI such as T1-weighted imaging (T1WI), T2-weighted imaging (T2WI), T1 contrast-enhanced imaging (T1CE), and fluid-attenuated inversion recovery (FLAIR) are clinically used for evaluating tumor morphology and anatomy. In recent years, advanced MRI techniques have become more available in clinical routines. Dynamic contrast-enhanced (DCE) MRI assesses tumor perfusion and permeability (11, 12). Diffusion tensor imaging (DTI) reflects tumor cell density and the integrity of white matter tracts (13, 14). DCE and DTI can offer complementary information on tumor anatomy, vascularity and microstructural organization, which indicate the biogenetic markers of the tumors.

Machine learning-based radiomics is the process of extracting high-throughput information from quantitative imaging data, analyzing and modeling (15). In recent years, radiomics has been proven promising in predicting key molecular markers in gliomas. However, most of these researches concentrated on conventional structural MRI (16–19). Currently, there is no research combining DCE and DTI radiomics in detecting MGMT methylation status. Whether adding DCE and DTI radiomic features can improve the model performance in pre-operative MGMT evaluation remains unclear. This study tries to address these gaps by exploring the potential of combining conventional structural MRI, DCE, and DTI-derived radiomic features for predicting MGMT methylation status in gliomas. Specifically, we seek to evaluate whether the inclusion of DCE and DTI features improves model performance over traditional structural MRI-based radiomics.

In this study, we aim to investigate the value of radiomic features derived from structural MRI, DCE, and DTI in predicting MGMT methylation status in glioma patients. We hypothesize that adding DCE and DTI radiomic features can increase the model performance in detecting MGMT promoter status.

## 2 Materials and methods

### 2.1 Study population

**Figure 1:**
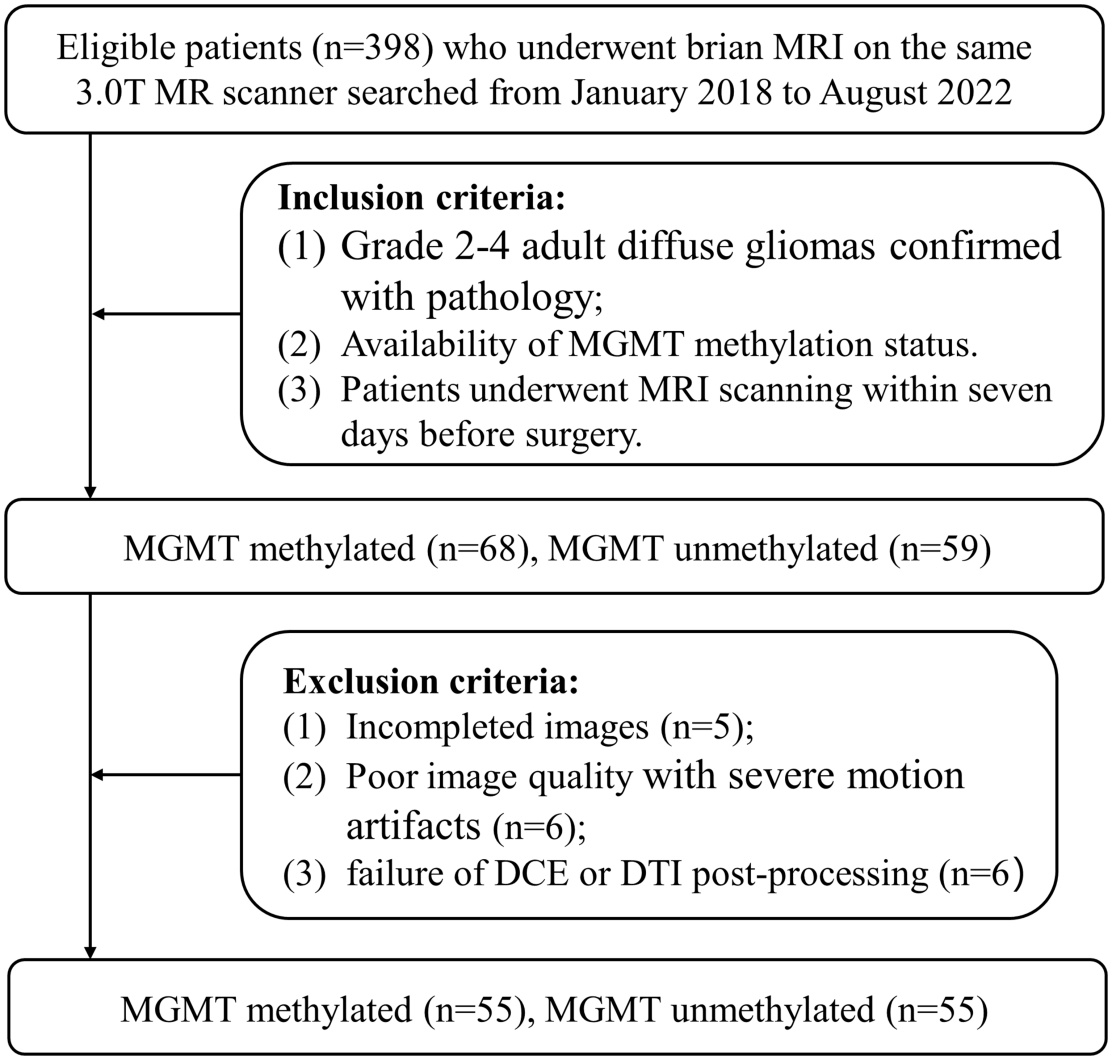
Patient enrollment flowchart. AUC = area under the curve; ACC= accuracy; SENS = sensitivity; PREC= precision; DCE = dynamic contrast enhanced;DTI=diffusion tensor imaging.

The protocol for this research was approved by our Institutional Ethics Committee (Approval Number: 2022-364-02) and performed in accordance with the Declaration of Helsinki. Patients in this research were enrolled in our hospital between January 2018 and August 2022. The requirement for informed consent was waived due to the retrospective nature of this research. The inclusion criteria were as follows: (1) grade 2–4 adult diffuse gliomas confirmed with pathology; (2) patients with known MGMT mutation status. (3) patients underwent MRI scanning within 7 days before surgery. The exclusion criteria were as follows: (1) incomplete images; (2) poor image quality with severe motion artifacts; (3) radiotherapy or chemotherapy before MRI scanning; (4) failure of DCE or DTI post-processing. According to the above criteria, a total of 110 patients were recruited in this research, of which 55 patients with MGMT methylated and 55 patients with MGMT unmethylated. MGMT promoter methylation status was tested using a quantitative polymerase chain reaction assay (see Figure 1).

### 2.2 MRI protocol

All the pre-operative MRI data were acquired using a 3.0 T MRI scanner (uMR790, United Imaging Healthcare, Shanghai, China) with a 32-channel phased-array head coil. Conventional MRI examinations included 3D T1WI pre- and post- the injection of gadolinium-based contrast agent [repetition time (TR)/echo time (TE) = 7.9/3.1 milliseconds; inversion time (TI) = 810 milliseconds; flip angle (FA) = 10°; matrix = 256 × 256; field of view (FOV) = 256 × 232 mm^2^; slice thickness = 1 mm], 3D T2WI (TR/TE = 2,200/606.36 milliseconds; TI = 1,519 milliseconds; FA: from 19° to 150°; matrix = 256 × 256; FOV = 256 × 232 mm^2^; slice thickness = 1 mm) and 3D FLAIR (TR/TE = 4,800/428.04 milliseconds; TI = 1,519 milliseconds; FA: from 21° to 150°; matrix = 240 × 240; FOV = 256 × 232 mm^2^; slice thickness = 1 mm).

DTI was performed with 32 directions and the following parameters: TR/TE =2,205/68.2 milliseconds; FA = 90°; matrix = 128 × 128; FOV = 230 × 230 mm^2^; slice thickness = 2 mm.

Axial DCE-MRI acquisition was performed using dynamic scan of a T1-gradient echo sequence and setting the following parameters: TR/TE = 3.47/1.9 milliseconds; FA = 13°; matrix = 160 × 160; FOV = 240 × 220 mm^2^; slice thickness = 5 mm. Pre-contrast images with multiple FA 5, 10 and 15° were acquired for the T1 maps. Then the contrast agent (Gadovist, 1 mmol/mL, Bayer Healthcare, Berlin, Germany) was administered (0.1 mmol/kg of bodyweight) through the antecubital vein via a power injector at a rate of 2 mL/s. A series of 1,800 images at 90 dynamic phases for 20 axial sections were obtained with a temporal resolution of 4 s for each dynamic phase.

### 2.3 DCE and DTI post-processing

United imaging software workstation was utilized to analyze DCE and DTI parameters. For DCE metrics analysis, the arterial input function was acquired with the ROI positioned in the internal carotid artery adjacent to the tumor side. The following perfusion parameters were analyzed based on the extended Tofts two-component model: Ktrans, Kep, Ve, Vp and iAUC. Ktrans refers to the volume transfer constant, signifying the flow of gadolinium from the blood plasma into the extravascular extracellular space (EES), Kep denotes the time constant of gadolinium reflux from the EES back into the vascular system, Ve represents the EES volume per unit tissue volume, Vp indicates the plasma volume per unit tissue volume, and iAUC illustrates to the initial area under the time-concentration curve for the first 60 s. For DTI metrics analysis, fractional anisotropy, mean diffusivity, axial diffusivity and radial diffusivity maps were calculated.

### 2.4 Image process and radiomic feature extraction

Each sequence was registered and resampled to a 1 × 1 × 1 mm voxel resolution represented by FLAIR image utilizing automated nonlinear registration. Subsequently, the resampled data was skull stripped. Intensity normalization was used to eliminate the greyscale distribution differences and made the MRI image histograms more consistent among patients.

**Figure 2:**
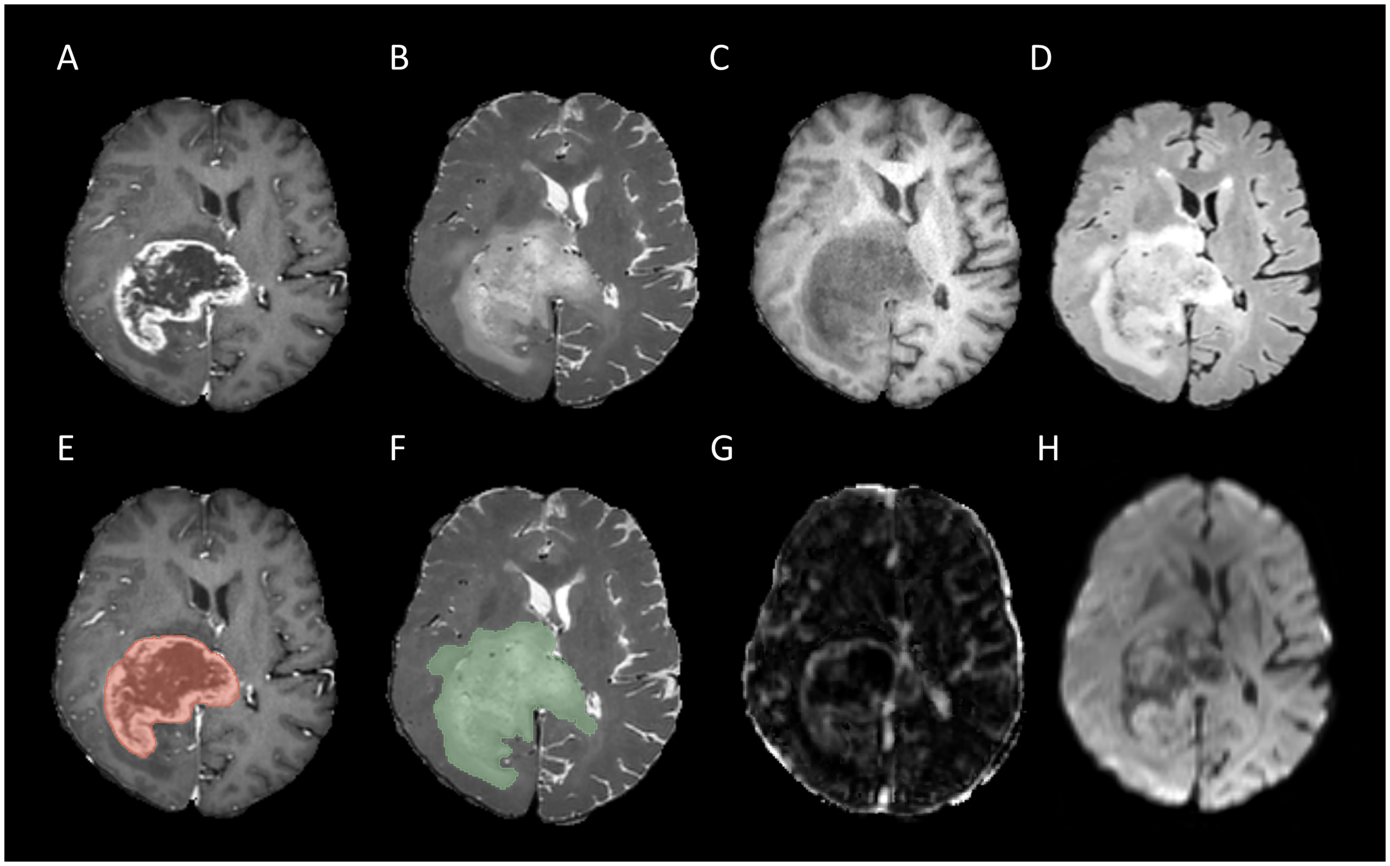
Example for MR images of a patient with MGMT unmethylated: T1CE **(A)**, T2WI **(B)**, T1WI **(C)**, FLAIR **(D)**, tumor core segmentation on T1CE **(E)**, whole tumor segmentation on T2WI **(F)**, Ktrans map of DCE **(G)**, and MD map of DTI **(H)**.

**Figure 3:**
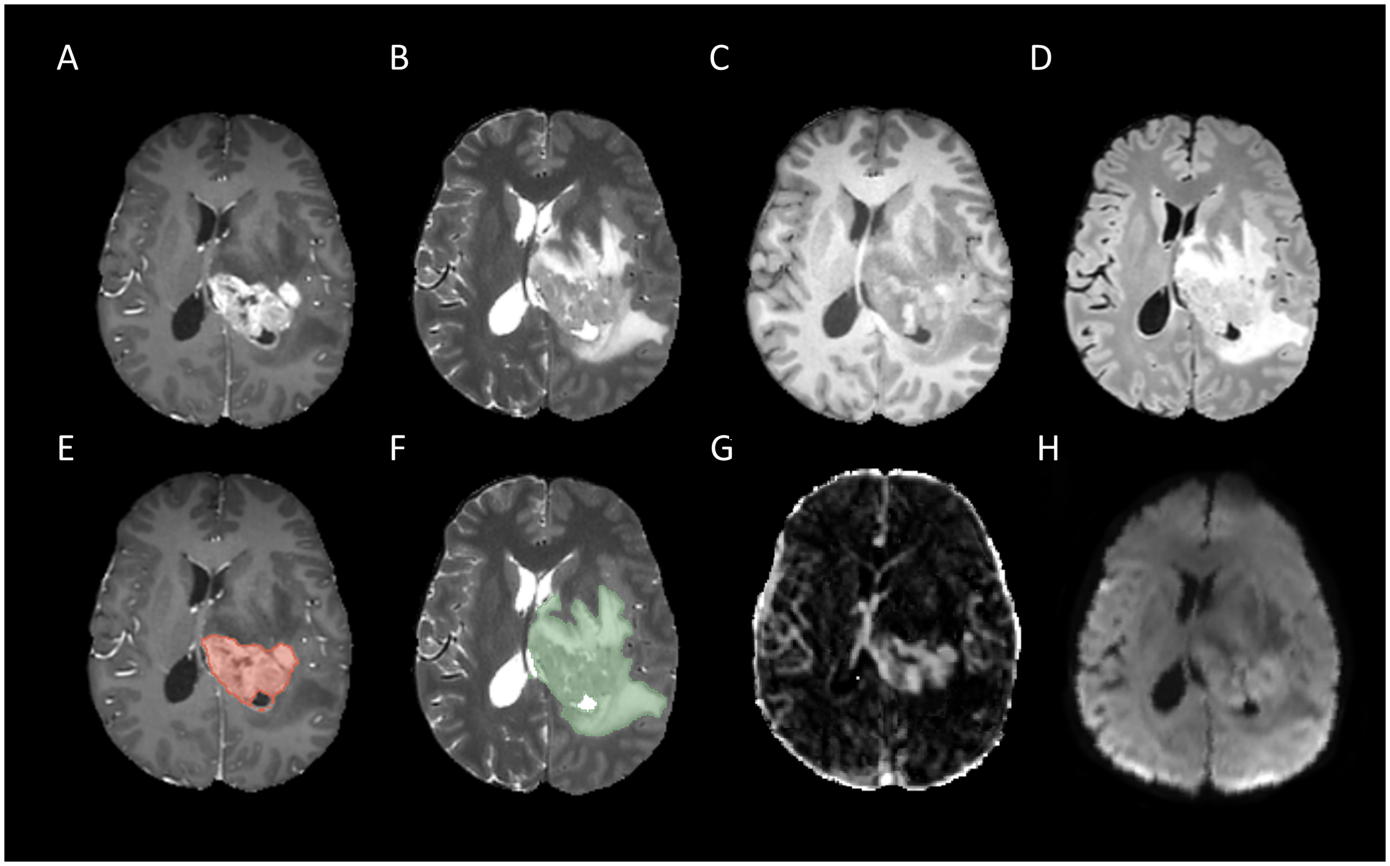
Example for MR images of a patient with MGMT methylated: T1CE **(A)**, T2WI **(B)**, T1WI **(C)**, FLAIR **(D)**, tumor core segmentation on T1CE **(E)**, whole tumor segmentation on T2WI **(F)**, Ktrans map of DCE **(G)**, and MD map of DTI **(H)**.

In this research, we focused on two key regions of interest (ROI): whole tumor (WT) and tumor core (TC). The WT refers to the entirety of the tumor, including the tumor core and the adjacent edematous regions, whereas the TC is specifically defined by regions of enhancing tumor tissue, non-enhancing tumor tissue, and necrotic areas. The segmentation of all corresponding labels was conducted manually by a board-certificated neuroradiologists with more than 15 years of experience in neuro-oncology. Two examples of tumor segmentation can be seen in Figures 2, 3.

Given that isotropic voxel dimensions can ensure the consistency of extracted features across all directions and thereby enhance the reliability of the analysis, we adopted the sitkBSpline interpolation method to resample the raw imaging data to an isotropic resolution of 1 × 1 × 1 mm^3^. For each patient, we selected four conventional structural MRI sequences (T1WI, T2WI, FLAIR, and TICE) alongside five DCE maps (iAUC90, Ktrans, Kep, Ve, and Vp) and DTI maps (FA, AD, MD, and RD), resulting in a total of 13 MRI sequences. For each sequence, we extracted features from two specific ROIs: TC and WT. A total of 107 features were obtained from each ROI, encompassing form shape features, first-order statistics, and texture features generated from various matrices such as gray-level co-occurrence matrices, size zone matrices, run length matrices, nearby gray tone difference matrices, and dependency matrices. One hundred and seven features were extracted from each of the 2 ROIs in the 13 sequences for each patient, for a total of 2,782 radiomic features.

### 2.5 Dataset splitting and *z*-score normalization

To maintain consistent data distribution between the training and test sets, we employed stratified sampling to randomly split the dataset into training and test datasets with an 8:2 ratio. Subsequently, we applied *z*-score normalization to the features of both the training and test sets, using the mean and standard deviation computed from the training set features.

### 2.6 Feature selection

The feature selection encompasses three meticulous steps: initially, we employ the Pearson correlation coefficient as a means of preliminary feature pre-selecting, establishing a threshold of 0.75 to discern and eliminate one member from each pair of features exhibiting a high degree of correlation (signified by an absolute correlation coefficient exceeding this threshold). Subsequently, we implement the least absolute shrinkage and selection operator (LASSO) regression model for granular feature selection. LASSO regression, through the incorporation of the L1 regularization term, exhibits an inherent capability to simultaneously select relevant features and shrink their corresponding coefficients. This process is optimized by leveraging GridSearchCV in conjunction with a five-fold cross-validation scheme, enabling us to determine the optimal regularization parameter *λ*. Finally, we harness the power of principal component analysis (PCA) for dimensionality reduction. PCA transforms the high-dimensional feature space derived from LASSO regression into a lower-dimensional space, specifically reducing it to 10 principal components. These principal components encapsulate the majority of the variance present in the original dataset, thereby preserving essential information while significantly simplifying the data representation.

### 2.7 Model development and evaluation

Based on the 13 MRI sequences, 7 sequence groups were selected: structural MRI, DCE, DTI, structural MRI + DCE, structural MRI + DTI, DCE + DTI, structural MRI + DCE+ DTI. Support vector machine (SVM), Gaussian naive bayes (GaussianNB), adaboost, logistic regression (LR), random forest and k-nearest neighbor (KNN) were utilized to establish multiple radiomics classification models, the area under the receiver operating curve (AUC), accuracy (ACC), sensitivity (SENS), precision (PREC) and *F*_1_ score were utilized to compare the performance of different radiomics models. The model with the highest AUC on the test set was selected as the final model. Decision curve analysis (DCA) was used to evaluate the clinical value of different radiomics models.

**Figure 4:**
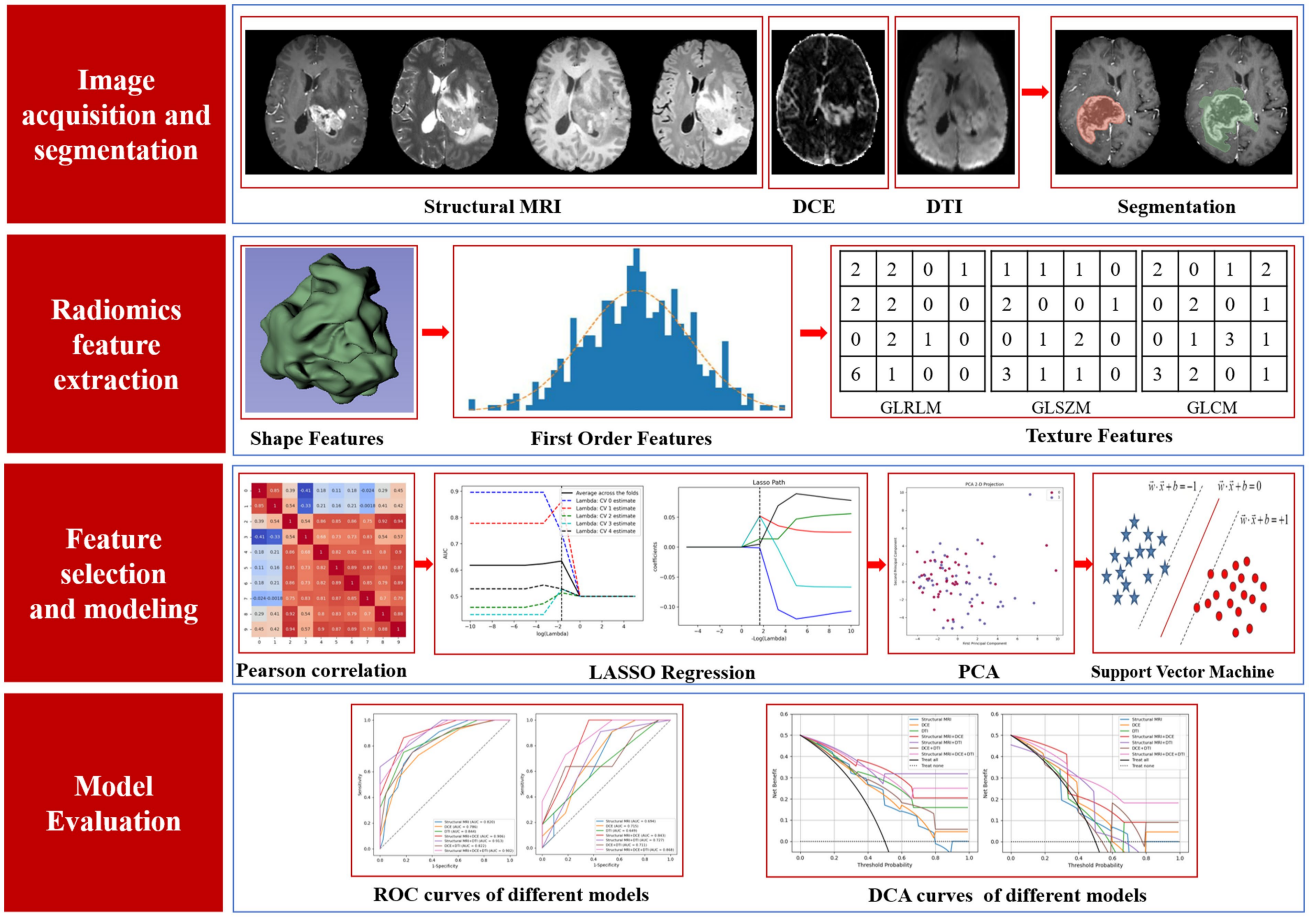
Study design of this research.

Two radiologists with 1 year and 5 years-experience in neuro-oncology imaging also evaluated MGMT promoter methylation status on the test dataset with all 13 sequences provided, as a comparison with multiple radiomics models. The study design of this research is shown in Figure 4.

### 2.8 Statistical analysis

To evaluate the normality of continuous variables, we implemented the Kolmogorov–Smirnov normality test. Independent samples *t*-test was used to compare the difference of continuous variables between the training dataset and the test set. The chi-square test was used to assess the distribution of categorical variables between groups, such as gender and MGMT promoter methylation, for categorical variables. In this study, a *p*-value of 0.05 or lower was considered statistically significant. Python 3.7.9^1^ was used to conduct the statistical analysis.

## 3 Results

### 3.1 Baseline information

Table 1 shows a brief summary of the baseline characteristics of the 110 patients who participated enrolled in this research. There were no significant differences in age, gender, or MGMT promoter methylation status between the training and test datasets. We used stratified sampling, which led to the identical distribution of MGMT promoter methylation status in both the training set and the test dataset.

**Table 1:** Characteristics of training dataset and test dataset.

Characteristics	Overall (*n* = 110)	Training (*n* = 88)	Test (*n* = 22)	*p*-value
Age (years)		52.841 ± 14.709	50.636 ± 12.579	0.520^a^
Sex				0.924^b^
Female	59 (53.6%)	47 (53.4%)	12 (54.5%)	
Male	51 (46.4%)	41 (46.6%)	10 (45.5%)	
MGMT				1.000^b^
Unmethylated	55 (50.0%)	44 (50.0%)	11 (50.0%)	
Methylated	55 (50.0%)	44 (50.0%)	11 (50.0%)	
WHO grade				0.746^b^
2	29 (26.4%)	24 (27.3%)	5 (22.8%)	
3	8 (7.3%)	7 (8.0%)	1 (4.5%)	
4	73 (66.3%)	57 (64.7%)	16 (72.7%)	

MGMT, O6-methylguanine-DNA methyltransferase.

a Independent samples *t*-test.

b Chi-squared test.

### 3.2 The performance of multiparametric MRI radiomics models to predict MGMT promoter methylation status of adult diffuse gliomas

**Figure 5:**
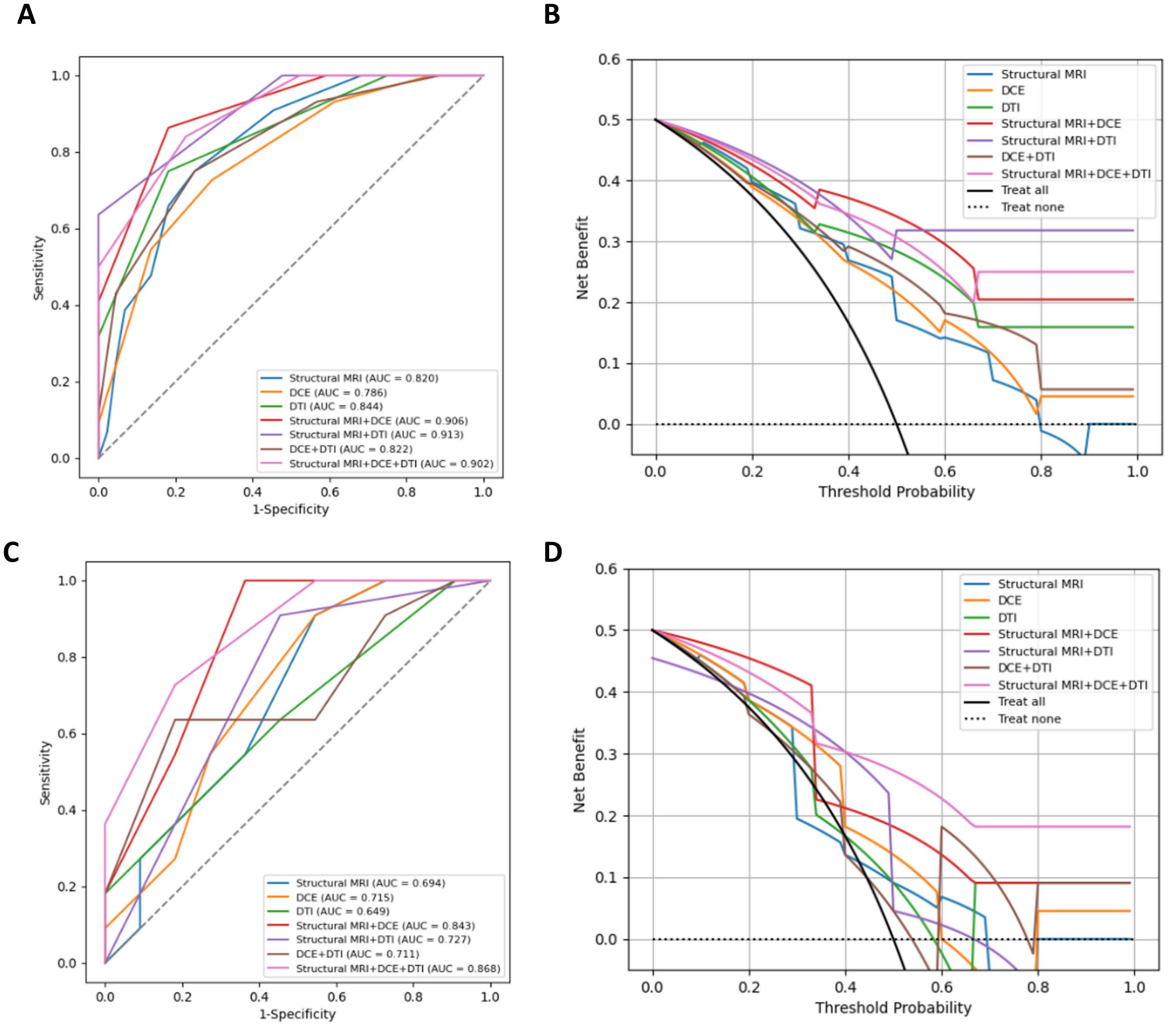
ROC curves, DAC curves for the training set **(A,B)** and test set **(C,D)** of different combined radiomics models in predicting MGMT methylation status.

On the training dataset, the modality combination structural MRI + DCE exhibited the highest performance with AUC of 0.906 and ACC of 0.841. The metrics of the multiparametric MRI radiomics models were illustrated in Table 2 and Figure 5. The model structural MRI + DCE also showed the highest clinical value on the DCA curve.

**Table 2:** Performance of different radiomics models to predict MGMT methylation status on training dataset.

Sequence group	AUC	ACC	SEN	PREC	*F*_1_ score
Structural MRI	0.820	0.671	0.477	0.778	0.592
DCE	0.786	0.716	0.727	0.711	0.720
DTI	0.844	0.784	0.750	0.805	0.777
Structural MRI + DCE	0.906	0.841	0.864	0.826	0.844
Structural MRI + DTI	0.913	0.818	0.636	1.000	0.778
DCE + DTI	0.822	0.750	0.750	0.750	0.750
Structural MRI + DCE + DTI	0.902	0.807	0.841	0.787	0.813

AUC, area under the curve; ACC, accuracy; SENS, sensitivity; PREC, precision; DCE, dynamic contrast enhanced; DTI, diffusion tensor imaging.

On the test dataset, the modality combination structural MRI + DCE + DTI exhibited the highest performance with AUC of 0.868 and ACC of 0.773. The metrics of the multiparametric MRI radiomics models were illustrated in Table 3 and Figure 5. The 1-year radiologist and 5-year radiologist achieved accuracy of 0.500 and 0.591, respectively. The combined model structural MRI + DCE + DTI also illustrated the highest clinical value on the DCA curve.

**Table 3:** Performance of different radiomics models and radiologists to predict MGMT methylation status on test dataset.

Sequence group	AUC	ACC	SEN	PREC	*F*_1_ score
Structural MRI	0.694	0.591	0.364	0.667	0.471
DCE	0.715	0.636	0.546	0.667	0.600
DTI	0.649	0.591	0.636	0.583	0.609
Structural MRI + DCE	0.843	0.682	0.546	0.750	0.632
Structural MRI + DTI	0.727	0.546	0.182	0.667	0.286
DCE + DTI	0.711	0.546	0.636	0.538	0.583
Structural MRI + DCE + DTI	0.868	0.773	0.773	0.800	0.762
1-year radiologist	—	0.500	0.546	0.500	0.522
5-year radiologist	—	0.591	0.545	0.600	0.571

AUC, area under the curve; ACC, accuracy; SENS, sensitivity; PREC, precision; DCE, dynamic contrast enhanced; DTI, diffusion tensor imaging.

The performance of different machine learning methods to predict MGMT methylation status, based on the combination of structural MRI, DCE, and DTI modalities was illustrated in Supplementary Table S1. The SVM model showed the best performance.

## 4 Discussion

In this research, we explored the application of radiomic features based on pre-operative multiparametric MRI to establish multiple radiomics models to predict the MGMT promoter methylation status of adult diffuse gliomas. The results showed that on the test dataset, The combined model structural MRI + DCE + DTI achieved the highest performance with an AUC of 0.868 and ACC of 0.773, outperforming two radiologists with 1-year and 5-year of experience. The constructed radiomics models exhibited excellent predictive performance in the MGMT methylation detection task, which indicated promising clinical application. We also found that DCE and DTI combined with conventional structural MRI radiomics significantly improved the accuracy of MGMT methylation status prediction compared to conventional structural MRI radiomics alone, which is expected to be applied in future clinical scenarios.

To our knowledge, this is the first study to investigate the value of combining multimodal radiomics features in the prediction of MGMT methylation status based on 13 sequences from conventional MRI, DCE, and DTI. In several previous studies, the value of features from structural MRI in predicting MGMT promoter methylation status was controversial. Kim et al. (20) evaluated 420 models trained with structural MRI from a publicly available dataset to detect MGMT promoter status, from which approximately 80% of models obtained no significant difference with the chance level of 50%. Another research conducted by Robinet et al. (21) explored different input configurations, algorithms as well as exact methylation percentages, and got the conclusion that current deep learning methods cannot determine MGMT promoter methylation status from merely structural MRI. In our study, the structural MRI model achieved an AUC of 0.694 and ACC of 0.591 on the test dataset, consistent with previous negative results, which further proved the limited effectiveness of structural MRI in detecting MGMT promoter methylation. The negative results pushed us to explore the value of other advanced MRI techniques like DCE and DTI in MGMT methylation prediction.

DCE is capable of quantitatively assessing vascular permeability, perfusion, and other hemodynamic parameters, such as Ktrans and Ve, by continuously monitoring the changes in signal intensity in tissues after intravenous injection of contrast agents (22). These parameters are critical for understanding tumor angiogenesis, assessing treatment efficacy and predicting disease progression (23). A study delved into the value of the application of DCE imaging technology in the assessment of MGMT promoter methylation status in glioblastoma (24). The results showed that GBM patients who developed MGMT methylated patients exhibited significantly higher Ktrans values compared to MGMT unmethylated, a finding that not only revealed a strong association between DCE imaging parameters and MGMT methylation status but also by setting Ktrans >0.086 as the optimal cut-off value, with an AUC of just 0.756. Another research explored the DCE histogram analysis of glioma and discovered that the 90th percentile Ve provided the highest differential efficacy for MGMT with an AUC of 0.816, sensitivity of 0.84, and specificity of 0.79 (25).

DTI is a non-invasive MRI technique providing quantitative information about the anisotropy and diffusivity of major white matter (26). Lots of studies have proven that DTI parameters FA and MD are associated with cellular physiology and tissue microstructure, which can reflect the genetic changes of tumors (27). In a previous study, researchers attempted to combine diffusion kurtosis imaging (DKI) and DTI modalities to build a model for predicting MGMT methylation status and revealed that adding DTI and DKI radiomics features cannot improve the performance for predicting MGMT methylation (28). Tan et al. (28) recruited 40 patients with insula gliomas, and showed that neither FA or MD histogram parameters predicted MGMT methylation status. In our study, on test dataset, the model structural MRI + DTI achieved an AUC of 0.727, while the model structural MRI achieved an AUC of 0.694. Adding DTI radiomics features only slightly improved the model performance, which is consistent with previous studies. While DTI provides valuable information on tissue anisotropy and diffusion patterns, the contribution of these features to predicting MGMT methylation may be relatively small compared to structural MRI and DCE parameters, which offer more direct information related to tumor morphology and vascularity. Moreover, the integration of multiple modalities, including structural MRI and DCE, could already encapsulate much of the relevant information, leaving less room for DTI features to contribute significantly.

Given the current inadequate performance demonstrated by a single modality in assessing the methylation status of the MGMT promoter, this study reflects deeply and recognizes that relying on the imaging data of one particular modality alone does have significant shortcomings in comprehensively capturing and resolving the complex brain tumor features (29, 30). We successfully revealed the significant advantages of multimodal data fusion in the prediction of MGMT methylation status by constructing and evaluating a combined multimodality imaging model including structural MRI, DCE, and DTI. Compared with methods relying on a single modality or any two-modality combination, the combined multimodal model was able to more accurately capture the complex relationship between data and reduce the risk of overfitting in the training process but also maintained a stable and significantly improved prediction performance in the test set validation, which fully verified the superiority of the multimodal data fusion strategy in improving the model reliability and prediction accuracy.

There were also several limitations in this study. First, this is a single-institutional retrospective study lacking independent external test dataset from other hospitals, introducing potential biases related to patient populations and imaging protocols, which may influence the reproducibility of the models in different hospitals and different imaging settings. Secondly, the small sample size, especially the relatively small size of the test dataset, limits the generalizability of the findings. We will employ larger, multi-canter cohorts to explore the generalizability in the future. Additionally, we chose the classic machine learning model SVM to conduct different experiments without incorporating more complicated deep learning structures like convolutional neural networks and vision transformers. Deep learning models offer advantages over traditional machine learning models, such as scalability, non-linearity and potentially higher accuracy. We will further investigate this research by employing deep learning models in the future.

## 5 Conclusion

In conclusion, we developed and validated radiomics models which obtained promising performance in predicting MGMT promoter methylation status. Adding DCE and DTI features can provide extra information to structural MRI in detecting MGMT promoter methylation.

## Data Availability

The raw data supporting the conclusions of this article will be made available by the authors, without undue reservation.

## Footnotes

^1^ http://www.python.org
