# Supplementary material for: Radiomics prediction of MGMT promoter methylation in adult diffuse gliomas: a combination of structural MRI, DCE, and DTI
Source: Front Neurol. 2025 Jan 29;16:1493666. doi: 10.3389/fneur.2025.1493666 (PMC11813925; doi:10.3389/fneur.2025.1493666)
Supplement: Supplementary file 1 [file Table_1.docx]

Supplementary Table 1. Performance of different machine learning methods to predict MGMT methylation status, based on the combination of Structural MRI, DCE, and DTI modalities.

| Cohort | Methods | AUC | ACC | SEN | PREC | F1 Score |
| --- | --- | --- | --- | --- | --- | --- |
| Training dataset | GaussianNB | 0.807 | 0.761 | 0.705 | 0.795 | 0.747 |
|  | Adaboost | 0.979 | 0.943 | 1.000 | 0.898 | 0.946 |
|  | LR | 0.767 | 0.727 | 0.727 | 0.727 | 0.727 |
|  | Random forest | 0.996 | 0.989 | 1.000 | 0.978 | 0.989 |
|  | KNN | 0.763 | 0.739 | 0.705 | 0.756 | 0.729 |
|  | SVM | 0.902 | 0.807 | 0.841 | 0.787 | 0.813 |
| Test dataset | GaussianNB | 0.752 | 0.682 | 0.727 | 0.667 | 0.696 |
|  | Adaboost | 0.500 | 0.455 | 0.727 | 0.471 | 0.571 |
|  | LR | 0.727 | 0.682 | 0.545 | 0.750 | 0.632 |
|  | Random forest | 0.814 | 0.773 | 0.727 | 0.800 | 0.762 |
|  | KNN | 0.727 | 0.682 | 0.636 | 0.700 | 0.667 |
|  | SVM | 0.868 | 0.773 | 0.773 | 0.800 | 0.762 |

AUC = area under the curve; ACC= accuracy; SENS= sensitivity; PREC= precision; GaussianNB= Gaussian Naive Bayes; LR= logistic regression; KNN= k-nearest neighbor; SVM= Support Vector Machine.
